# Lung resident mesenchymal cells isolated from patients with the Bronchiolitis Obliterans Syndrome display a deregulated epigenetic profile

**DOI:** 10.1038/s41598-018-29504-5

**Published:** 2018-07-24

**Authors:** Serena Vella, Pier Giulio Conaldi, Emanuela Cova, Federica Meloni, Rosa Liotta, Salvatore Cuzzocrea, Lavinia Martino, Alessandro Bertani, Angelo Luca, Patrizio Vitulo

**Affiliations:** 1 Department of Laboratory Medicine and Advanced Biotechnologies, IRCCS-ISMETT (Istituto Mediterraneo per i Trapianti e Terapie ad Alta Specializzazione), Palermo, Italy; 2 Department of Respiratory Diseases, IRCCS San Matteo Foundation and University of Pavia, Pavia, Italy; 3 Department of Diagnostic and Therapeutic Services, Pathology Service, IRCCS-ISMETT (Istituto Mediterraneo per i Trapianti e Terapie ad Alta Specializzazione), Palermo, Italy; 4 Department of Chemical, Biological, Pharmaceutical and Environmental Sciences, University of Messina, Messina, Italy; 5 Department for the Treatment and Study of Cardiothoracic Diseases and Cardiothoracic Transplantation, IRCCS-ISMETT (Istituto Mediterraneo per i Trapianti e Terapie ad Alta Specializzazione), Palermo, Italy; 6 Department of Diagnostic and Therapeutic Services, Radiology Service, IRCCS-ISMETT (Istituto Mediterraneo per i Trapianti e Terapie ad Alta Specializzazione), Palermo, Italy; 7 Present Address: Anemocyte S.r.l, Gerenzano, Italy

## Abstract

Bronchiolitis Obliterans Syndrome is the major determinant of the graft function loss after lung transplantation, but its pathogenesis is still incompletely understood and currently available therapeutic strategies are poorly effective. A deeper understanding of its pathogenic mechanisms is crucial for the development of new strategies to prevent and treat this devastating complication. In this study, we focused on the mesenchymal stromal cells, recently recognized as BOS key effectors, and our primary aim was to identify their epigenetic determinants, such as histone modifications and non-coding RNA regulation, which could contribute to their differentiation in myofibroblasts. Interestingly, we identified a deregulated expression of histone deacetylases and methyltransferases, and a microRNA-epigenetic regulatory network, which could represent novel targets for anti-fibrotic therapy. We validated our results *in vitro*, in a cell model of fibrogenesis, confirming the epigenetic involvement in this process and paving the way for a new application for epigenetic drugs.

## Introduction

Lung transplantation (LTx) is the most effective treatment option for end-stage lung disease patients^1^. Despite advances in this field, the chronic allograft rejection continues to be a major problem, largely due to the development of chronic rejection, whose main phenotype is represented by Bronchiolitis Obliterans Syndrome (BOS)^1^.

BOS is characterized by inflammatory/fibrotic processes inducing the partial or complete luminal occlusion of small airways^2^. It affects approximately 50% of lung transplant recipients (LTRs) by 5 years and 76% by 10 years^1^, with scarce effective treatments against it^2^. Although several risk factors have been associated with its onset^2^, the specific pathogenic mechanisms remain unknown and key mediators of airway injury and fibrosis need to be identified.

Recently, significant attention has been paid to multipotent donor-derived lung mesenchymal stem cells (MSC), isolated from the bronchoalveolar lavage fluid (BALf) of LTRs. MSC have been considered the primarily responsible for fibrous obliteration of small airways, since they proliferate more than those isolated from stable lung transplant recipients and had a characteristic profibrotic phenotype *in vitro*^3–5^. Several studies have been demonstrated a significant increase of MSC in the BALf from BOS patients^5–7^, suggesting them as BOS predictors, although their specific pathogenic role remains to be evaluated.

Global change in gene expression in MSC contributes to their differentiation in myofibroblasts, responsible of extracellular matrix remodeling and collagen deposition, which finally give rise to small airways obliteration^8^.

Epigenetics and microRNAs (miRNAs) are the two main mechanisms involved in the regulation of gene expression, crucial for mammalian development and cell differentiation, and their deregulation causes human diseases^9^, including lung fibrosis^8,10,11^.

Interestingly, epigenetics also controls miRNA expression, forming an epigenetics–miRNA regulatory circuit. To our knowledge, this network in BOS has not been studied yet.

In this study, we explored epigenetic and miRNA signature of lung MSC isolated from BALf of BOS patients, which could contribute to their differentiation in myofibroblasts, to fibroproliferative process and, ultimately, to BOS onset and progression. In addition, to further corroborate our results, we evaluated the epigenetic involvement in an *in vitro* cell model of fibrogenesis, blocking histone deacetylation.

## Results

### MSC from BALf of BOS patients differentially express epigenetic enzymes

To study the epigenetic profile of BOS effectors, MSC were isolated from 10 BALf of LTR: 5 BOS patients (2 BOS 0p and 3 established BOS grade 1–3) and 5 LTRswith stable lung function (Supplementary Table S1).

At the first culture passage, MSC expressed stem cell markers such as CD44, CD105, CD90, in the absence of hematopoietic markers (data not shown). Moreover, they had a high expression of FOXF1, a lung mesenchymal marker^7^, particularly those isolated from BOS patients’ BALf (data not shown).

We extracted RNA from MSC at low passages (between 2^nd^ to 6^th^) and analyzed by qRT-PCR the expression of several human epigenetic genes: 84 genes were analysed using arrays and 2 genes using single TaqMan assays (for MepC2 and EZH2).

**Figure 1:**
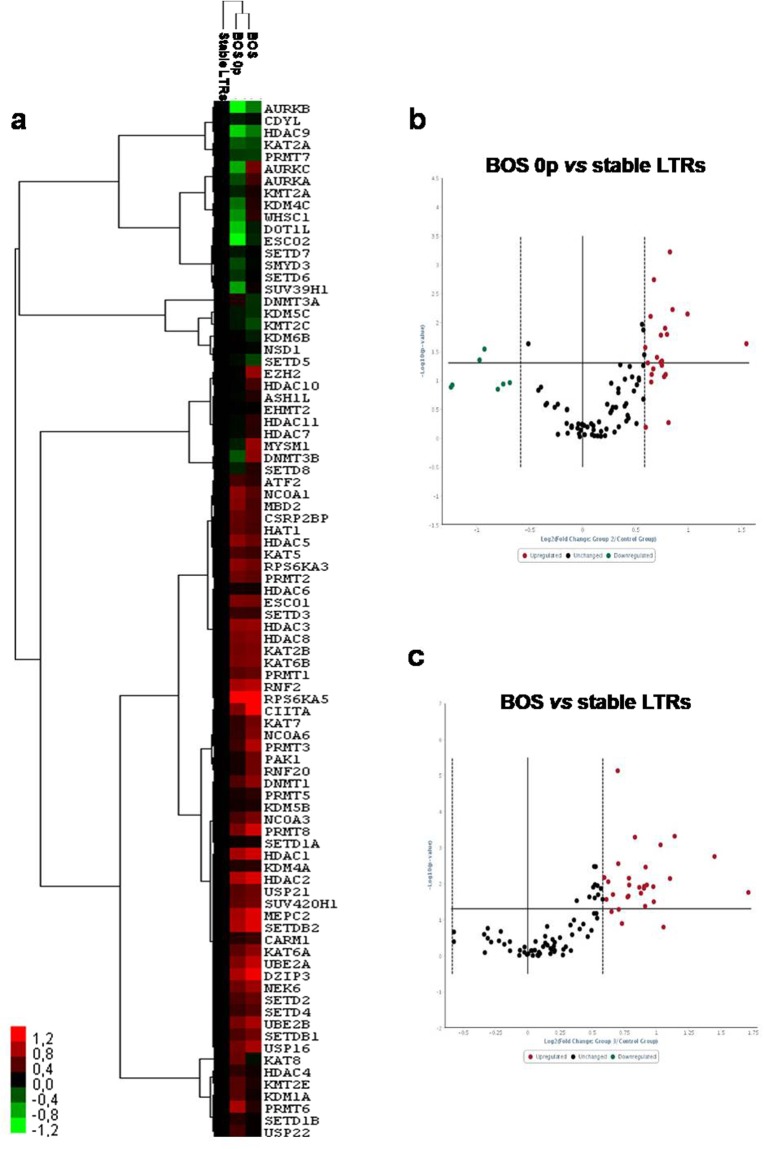
MSC from BALf of BOS patients differentially express epigenetic enzymes respect to those of stable LTRs. (**a**) Non-supervised hierarchical clustering based on the gene expression levels in MSC from BALf of stable LTRs, BOS 0p and BOS (n = 5, n = 2, n = 3, respectively). The dendrogram shows the relationships among gene expression patterns: “red” indicates high relative expression, “black” no change, and “green” low relative expression. Volcano plots showing differentially expressed genes between BOS 0p *vs* stable LTRs (**b**), and BOS *vs* stable LTRs (**c**). The volcano plot displays statistical significance versus fold-change on the y- and x-axes, respectively. A total of 86 genes were analysed. 17 genes have significantly different expression in MSC from BOS 0p samples respect to controls (p ≤ 0.05): 15 with fold change ≥1.5, and 2 with fold change ≤0.5. Twenty-three genes were significantly upregulated (fold change ≥ 1.5), when comparing the mRNA expression levels between MSC from stable LTRs and BOS samples.

Hierarchical clustering analysis confirmed the relationships among samples and showed systematic variations in the gene expression among the different groups (Fig. 1a). Interestingly, MSC from BOS (n = 3) had similar epigenetic gene expression to those of BOS 0p samples (n = 2), and both were very different from stable LTRs (n = 5) (Fig. 1a).

Among the epigenetic genes analysed, we identified: 15 significantly upregulated mRNAs (Supplementary Table S2) and 2 downregulated (Supplementary Table S3) in MSC from BOS 0p samples respect to controls (Fig. 1b); while 23 mRNAs were significantly upregulated, when comparing the mRNA expression levels between MSC from BOS samples and stable LTRs (Supplementary Table S4, Fig. 1c).

In addition, we randomly selected five genes (DNMT1, DNMT3A, HAT1, HDAC1 and HDAC4) to verify the array results, and validated their expression levels by qRT-PCR using single TaqMan assays. The qRT-PCR data were consistently in agreement with the RT^2^ array results (Supplementary Fig. S1a,b).

In particular, among the 23 most upregulated mRNAs in MSC from BOS patients (n = 3) respect to stable LTRs (n = 5), we identified two over-represented classes of epigenetic enzymes: histone deacetylases class I (HDAC1, HDAC2, HDAC3 and HDAC8) and methyltransferases (DNMT1, DNMT3B and EZH2).

**Figure 2:**
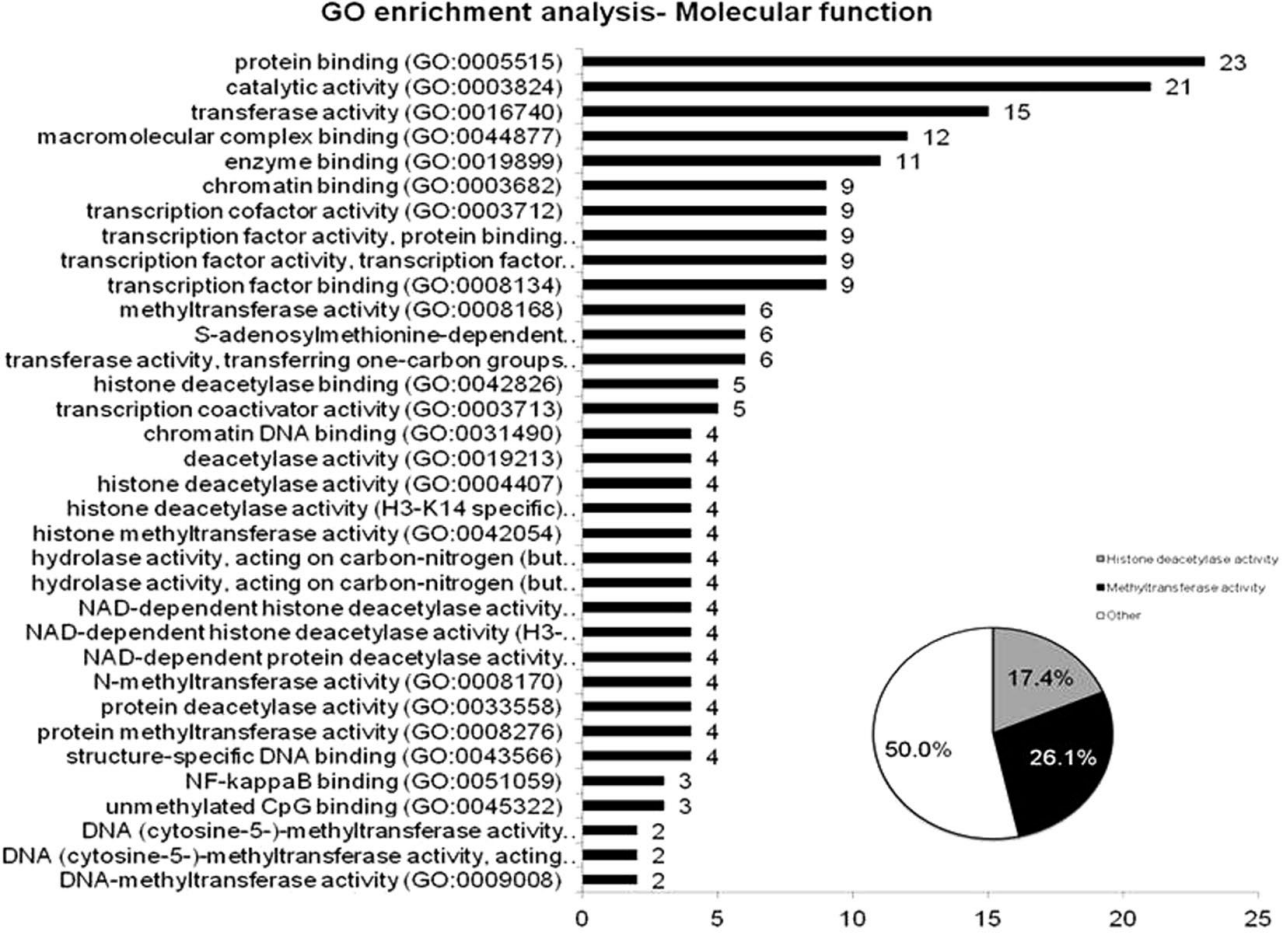
MSC from BALf of BOS patients present a deregulated expression of histone deacetylases and methyltransferases, and a pro-fibrotic phenotype. Functional categorization of upregulated genes in MSC of BOS samples based on gene ontology (GO) annotations.

Furthermore, using PANTHER Functional Annotation Chart, the 23 upregulated genes in MSC from BOS were grouped manually into ontology classes according their known or predicted molecular functions defined in Gene Ontology Consortium. Notably, the result confirmed that the most significant functional groups consisted of enzymes with histone deacetylase and methyltransferase activities (Fig. 2; Supplementary Tables S5 and S6).

To validate the expression data, as the histone deacetylases class I was the most over-represented epigenetic enzymes identified in MSC of BOS patients and, in particular, among these, HDAC1 was the most highly expressed, we analysed its expression in lung tissues from patients with BOS (n = 4) and stable LTRs (n = 4). QRT-PCR data on the whole lung extracts showed that BOS patients had a higher expression of HDAC1 (p = 0.04) (Supplementary Fig. S2a), confirming data on MSC from BALf.

Moreover, biopsies of BOS patients highly expressed FOXF1 which is correlated with the number of MSCs detected in BALf and with the presence of myofibroblasts in fibrotic lesions of lung allografts^7^ (data not shown).

### Profibrotic gene expression changes in MSC from BOS patients

Since accumulating evidence demonstrates that epigenetic modificationoccurs in fibroblast activation and fibrogenesis^12^, influencing the expressionof pro- and anti-fibrotic genes, we analysed by qRT-PCR the expression levels of some genes related to fibrosis, focusing on MSC from BOS patients. Interestingly, they had a slight increase of profibrotic genes (FN, Col1A1 and CTGF) and a decrease of PPARγ and CD90 (Supplementary Fig. S3a), although the differences were not statistically significant (probably due to the small sample size).

These results suggest that MSC from BALf of BOS patients seemmore prone to differentiate in myofibroblasts, responsible of collagen deposition and finally of small airways obliteration, as previously demonstrated^7^, and that this predisposition could be attributedin particular to epigenetic involvement.

### MiRNAs were differentially expressed by MSC from BALf of BOS patients respect to those of stable LTRs

To study the miRNA expression profile of MSC from BALf, we analyzed their miRNome by TaqMan Low Density Arrays (TLDA cards).

**Figure 3:**
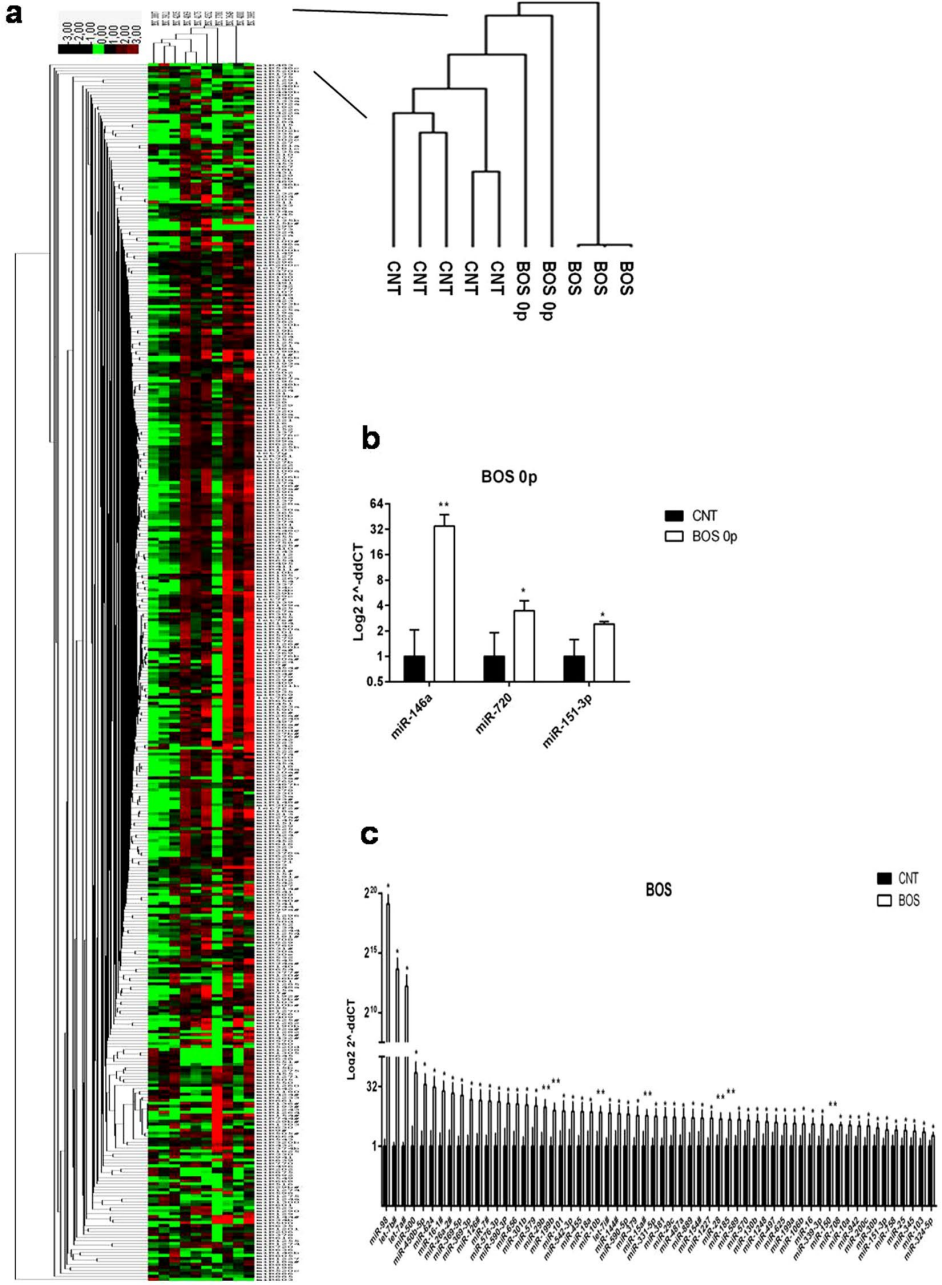
Differentially expressed miRNAs in MSC from BALf of BOS, BOS 0p and stable LTRs. (**a**) Non-supervised hierarchical clustering based on the miRNA expression levels in MSC from BALf of stable LTRs, BOS 0p and BOS (n = 5, n = 2, n = 3, respectively). Cluster 3.0 and Treeview softwares were used. The dendrogram shows the relationships among miRNA expression patterns. Red colour indicates upregulated genes, black colour downregulated genes and green colour indicates no change. A total of 754 miRNAs were analysed. 3 miRNAs are up-regulated in MSC from BOS 0p samples respect to controls (fold change ≥ 1.5; p ≤ 0.05); (**b**) 60 miRNAs were significantly upregulated (fold change ≥ 1.5; p ≤ 0.05), when comparing the miRNA expression levels between MSC from BOS samples respect to stable LTRs (**c**). Data are expressed as Mean (log_2_ scale) (±SD). *p ≤ 0.05; **p ≤ 0.001, two-tailed Student’s test.

Interestingly, hierarchical clustering analysis confirmed the relationships among samples and showed systematic variations in the miRNA expression among the groups (Fig. 3a).

Among the 754 miRNAs analysed, we found 409 miRNAs expressed in all the samples, with different expression levels.

We identified 3 significantly upregulated miRNAs in BOS 0p samples (n = 2; Fig. 3b), and 60 significantly upregulated miRNAs in BOS_1–3_ samples (n = 3) respect to stable LTRs (n = 5) (Fig. 3c). No significant downregulated miRNAs were found (Supplementary Table S7).

To verify the cards results, we validated the expression levels of five miRNAs (miR-106a, miR-124, miR-146a, miR-18b and miR-372), randomly selected, by qRT-PCR TaqMan single assays (Supplementary Fig. S1c,d).

In particular, in MSC from BOS patients, we identified a general increase of pro-fibrotic mediators (such as miR-199 family, miR-142-3p, miR-146a, miR-155, miR-17/92 cluster, miR-18, miR-192, miR-21, miR-224, miR-23a/27a/24 cluster, miR-451, miR-449, miR-7, miR-15/107 family, miR-365 miR-101, miR-144) and a decrease of anti-fibrotic miRNAs (miR-145, miR-206, miR-125b, let-7c).

In addition, we analyzed the bidirectional relationship between miRNAs and epigenetics. We found downregulated several epigenetically silenced miRNAs (miR-124a, miR-338-3p, miR-302b, miR-372, miR-520b, miR-523, miR-603, miR-136, miR-516-3p and miR-129). We also highlighted the downregulation of some miRNAs (miR-483-5p, 1264, 302/367 clusters and 371 cluster) which arekey mediators of epigenetic regulation.

To further corroborate our results, we evaluated the expression of five miRNAs (miR-106a, miR-124, miR-146a, miR-18b and miR-372) in lung tissues from patients with BOS (n = 4) and stable LTRs (n = 4). Supplementary Fig. S2b,c showed that trends for up-and down-regulation of these miRNAs were consistent, confirming the qRT-PCR data of MSC isolated from BOS patients’ BALf.

### SAHA inhibits TGF-β1-induced transition of MRC5 into myofibroblasts

Since our results highlighted an elevated expression levels of histone deacetylase enzymes class I, we supposed that this particular epigenetic process could have a central role in fibrogenesis.

We used fetal lung fibroblasts (MRC5) treated with TGF-β1 as *in vitro* cell model of fibrosis and took advantage to an histone deacetylase inhibitor, suberoylanilide hydroxamic acid (SAHA).

We chose human MRC-5 cells as representative lines for studying changes during lung fibrosis because this cell line has been validated previously as *in vitro* model of lung fibrosis^13–18^.

To evaluate whether SAHA could inhibit the TGF-β1-induced proliferation and differentiation into myofibroblasts, MRC5 were treated either with TGF-β1, SAHA or both.

**Figure 4:**
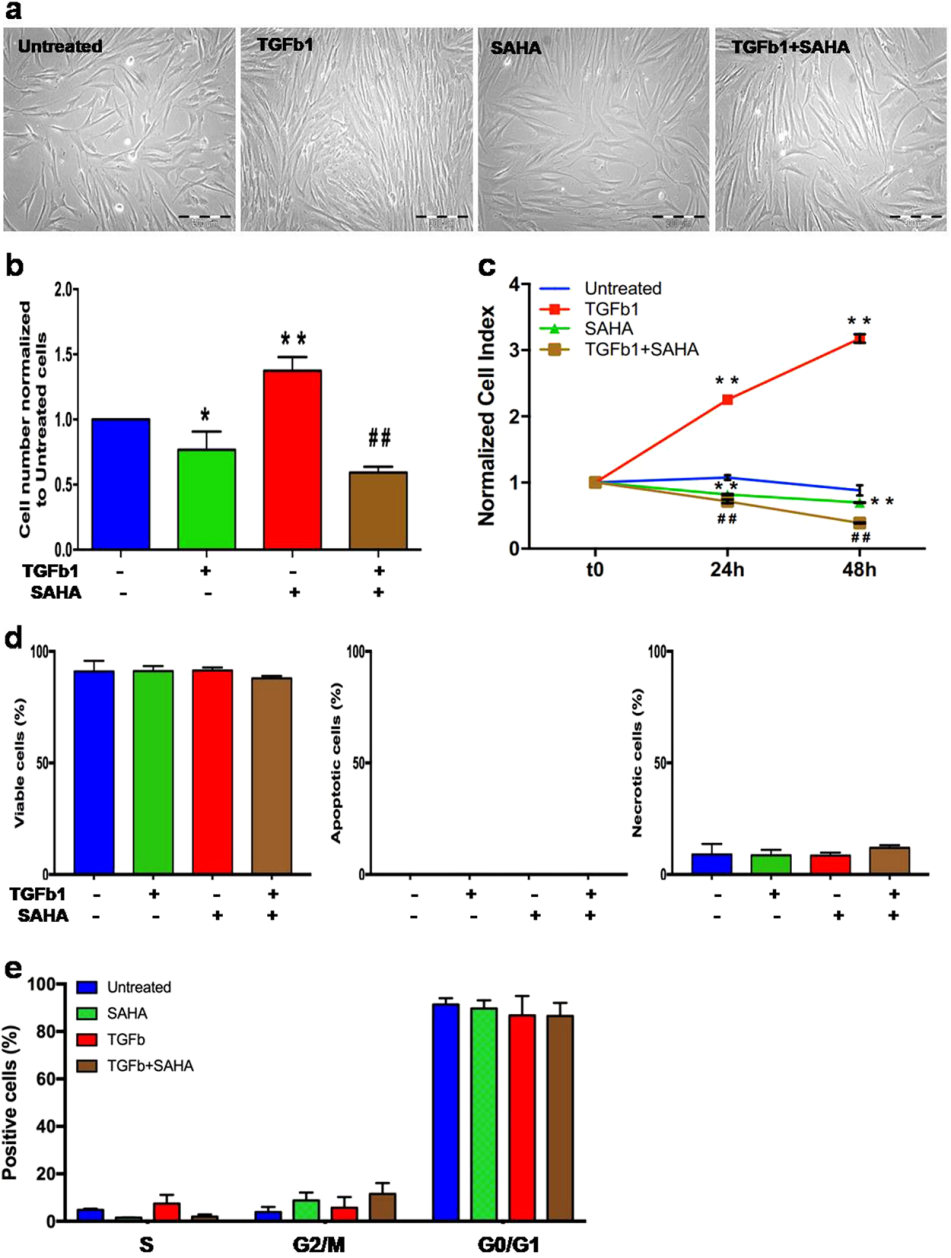
SAHA inhibits TGF-β1-induced cell proliferation. (**a**) Phase-contrast photographs of MRC5 treated with TGF-β1 and/or SAHA for 24 h (10x magnification). The experiment was performed three times (in triplicate per condition). (**b**) Cell proliferation of MRC5 was evaluated by cell countingassay. Results are presented as the mean number ± SD of MRC5 at 24 hours of incubation for each condition. The same amount of cells was used to start each incubation time, control and treated cells. Three independent experiments were carried out with three replicatesfor each experimental condition. One star (*) indicates a p ≤ 0.05, while two stars (**) indicate a p ≤ 0.001, compared to control group; and ^##^ indicates a p ≤ 0.01, compared to TGF-β1-treated group, two-tailed Student’s test. (**c**) Effect of TGF-β1 and/or SAHA on the viability of MRC5 was measured based on cell index by the xCELLigence system (Roche). Cell Index (CI) was recorded every 30 minutes. Each trace at each concentration was an average of 3 replicates. Data are normalized to the time of treatments addition to cell cultures. One star (*) indicates a p ≤ 0.05, while two stars (**) indicate a p ≤ 0.001, compared to control group; and ^## ^indicates a p ≤ 0.01, compared to TGF-β1-treated group, two-tailed Student’s test. (**d**) Apoptotic/necroticeffects of the different treatment on MRC5 were evaluated by Annexin V/PI staining. Graphs showing the percentage of viable, apoptotic and necrotic cells treated SAHA and/or TGF-β1 for 24 h. The experiment was performed three times. (**e**) Effect of TGF-β1 and/or SAHA on the cell cycle of MRC5 cells. After 24 h of treatment, cells were stained with propidium iodide to determine the DNA content. The percentages of the cells in each phase of the cell cycle were determined by flow cytometric analysis. The experiment was performed three times.

As shown in Fig. 4a, TGF-β1 changed cellular morphology inducing hypertrophy in MRC5, as previously described^19^, but, in presence of SAHA, this TGF-β1-induced morphological change was less evident.

Moreover, as TGF-β1 is known to influence cell proliferation, we performed cell counting and observed that SAHA alone reduced cell number compared to untreated cells (p ≤ 0.05), but, more interestingly, this drug significantly suppressed TGF-β1-induced MRC5 cell proliferation after 24 h of treatment (p ≤ 0.01 *vs* untreated cells and *vs* TGB1-treated cells) (Fig. 4b). These data were confirmed by the real-time xCELLigence system, which permitted us to followed cell proliferation at 24 and 48 hours (p ≤ 0.01 *vs* untreated cells and *vs* TGB1-treated cells, at each time point) (Fig. 4c).

Since the decreased cell number could be due to an increased mortality rather than the inhibition of cell proliferation, we performed a flowcytometric determination of apoptosis/necrosis by annexin V/propidium iodide double staining.

As shown in Fig. 4d and Supplementary Fig. S4, the percentages of apoptotic or necrotic or viable cells were similar among the different treatment groups, confirming that SAHA is not a pro-apoptotic drug as previous demonstrated by Wang *et al*.^20^.

Then, we investigated drug effects on cell-cycle progression, to determine whether the cell number reduction in SAHA/TGF-β1-treated MRC5 is due to the altered cell cycle distribution.

We demonstrated, by FACS analysis, that TGF-β1 induced a slight increase of S and a small reduction of G2/M phase population in MRC5 cell lines, when compared to untreated cells. On the contrary, these effects were partially restored by SAHA. Indeed, the combination of SAHA and TGF-β1 presented a decreased percentage of cells with S phase DNA content and an accumulation of cells in G2 phase, compared to TGF-β1-treated cells (Fig. 4e and Supplementary Fig. S5).

Differences in the G0/G1 phase were not significant among the several treatment groups (Fig. 4e).

Altogether these data suggest that SAHA reduced theTGF-β1effect on MRC5 proliferation.

**Figure 5:**
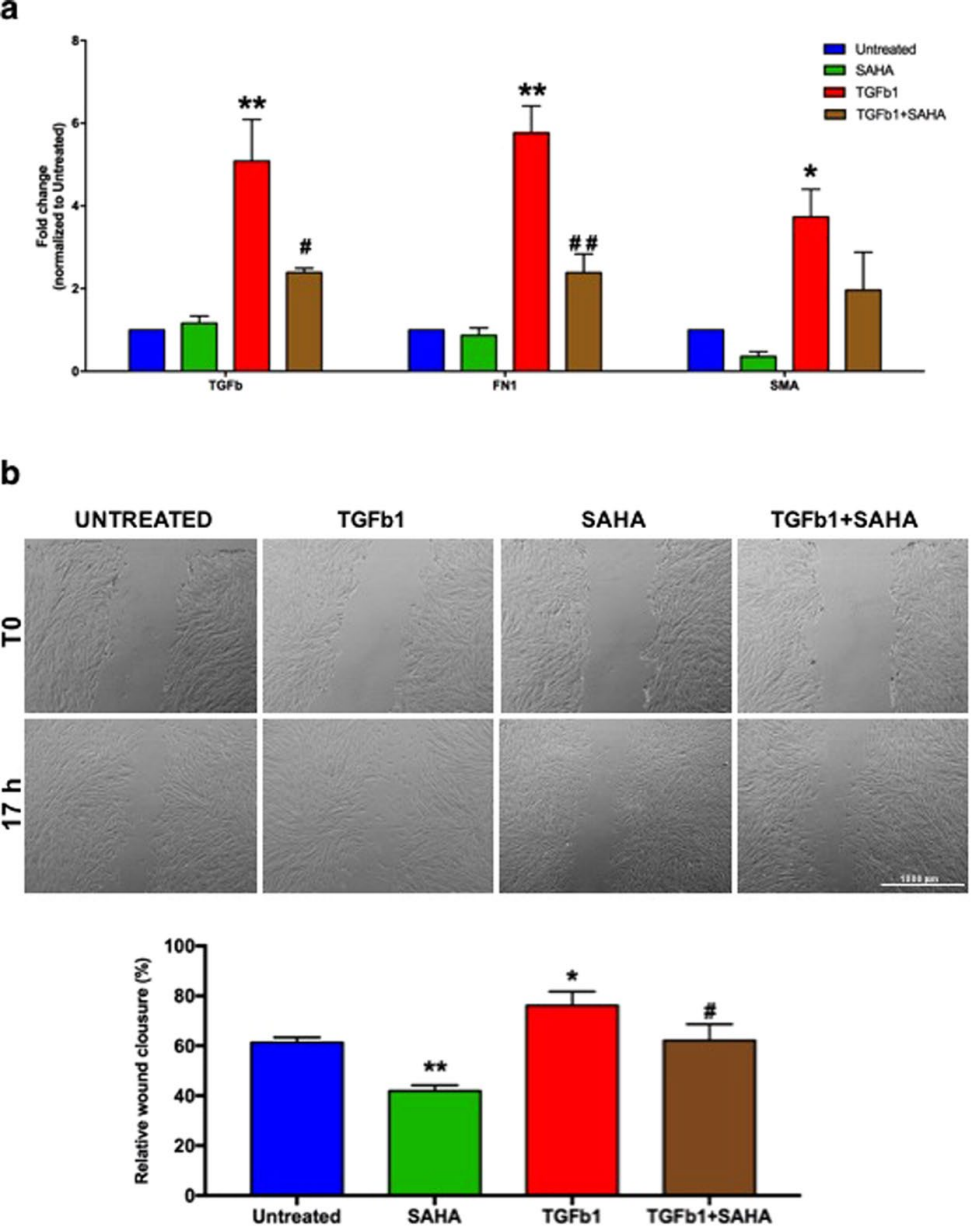
SAHA hinders TGF-β1-induced cell differentiation into myofibroblasts. (**a**) Quantitative RT-PCR results of pro-fibrotic genes (TGF-β1, FN1, aSMA) in MRC5 treated with or without SAHA and/or TGF-β1. Data are expressed as Mean (±SD). Three independent experiments were carried out with three replicatesfor each experimental condition. (**b**) Graphical representation of wound healing assay (magnification 10x). Data were obtained by two different observers. Results shown are the mean of three independent experiments ± SD (two replicates for each treatment). One star (*) indicates a p ≤ 0.05, while two stars (**) indicate a p ≤ 0.01, compared to control group; ^#^ and ^## ^indicate a p ≤ 0.05, or p ≤ 0.01, respectively, compared to TGF-β1-treated group, two-tailed Student’s test.

Next, to evaluate whether SAHA could also inhibit TGF-β1-driven differentiation, we analyzed by qRT-PCR many markers of lung fibrosis, such as TGF-β1, aSMA and FN1. As expected, TGF-β1 was able to induce high levels of these markers (p ≤ 0.01), but their expression was reduced in presence of SAHA. Interestingly, the treatment with SAHA decreased aSMA expression compared to untreated MRC5, suggesting that this drug could influenceboth basal and TGF-β1-induced expression of aSMA (Fig. 5a).

As TGF-β1 is involved in fibroblast migration^21^, we performed cell scratch assays for evaluating cell migratory capacity under different treatments.

We observed that TGF-β1 treatment significantly increased the migration capacity of MRC5 (as expected) (p = 0.0104 *vs* untreated cells), while the combination with SAHA decreased TGF-β1-induced migration (p = 0.014 *vs* TGF-β1-treated cells) (Fig. 5b).

In addition, we evaluated the expression levels of some epigenetic genes, such as HDAC1, DNMT1, EZH2 and MecP2, which could have a role in fibrogenesis, as we supposed.

**Figure 6:**
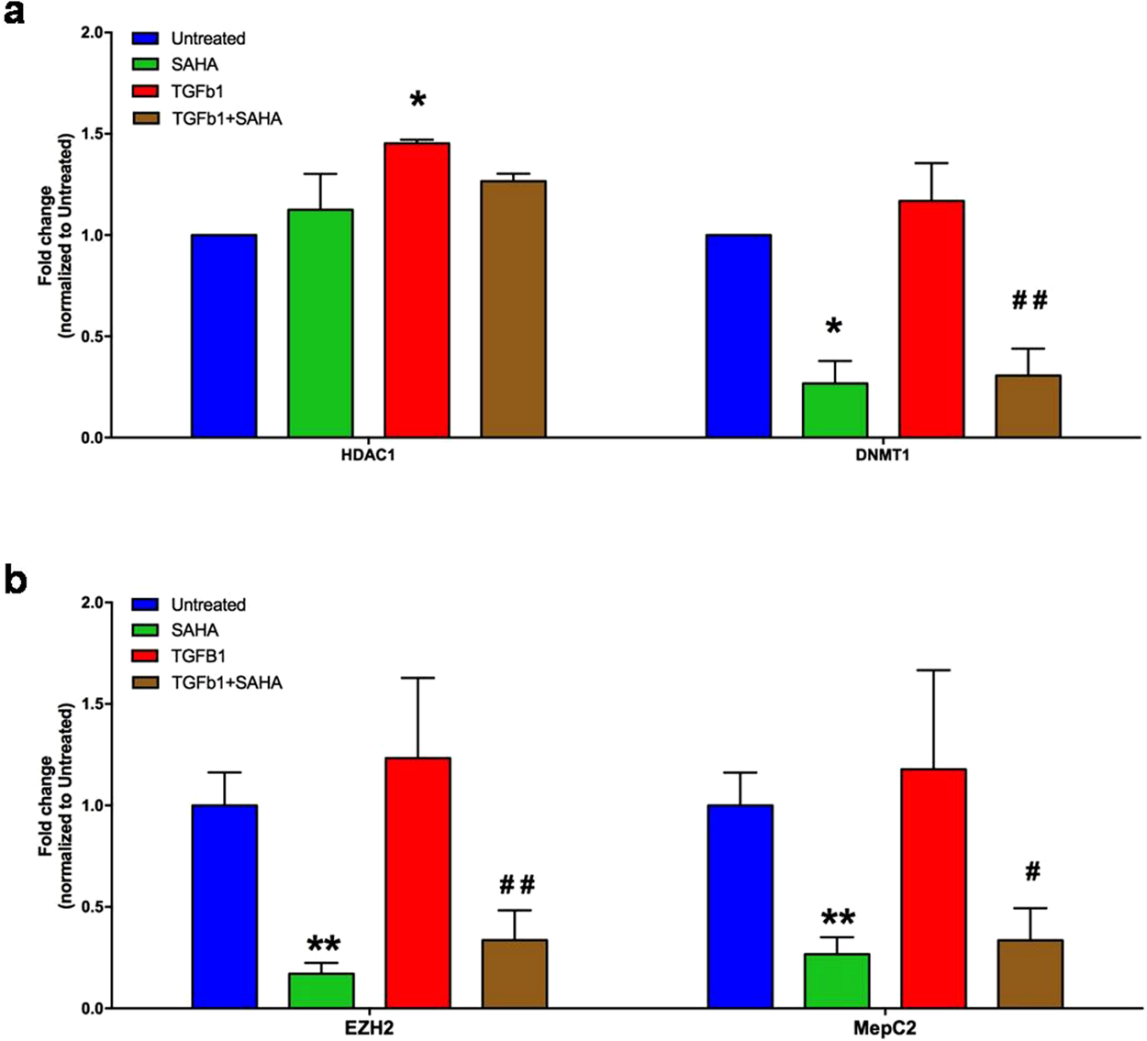
SAHA influences epigenetic gene expression. Quantitative RT-PCR results of HDAC1 and DNMT1 (**a**), EZH2 and MepC2 (**b**) in MRC5 treated with or without SAHA and/or TGF-β1. Data are expressed as Mean (±SD). One star (*) indicates a p ≤ 0.05, while two stars (**) indicate a p ≤ 0.01, compared to control group; ^#^ and ^## ^indicate a p ≤ 0.05 or p ≤ 0.01, respectively, compared to TGF-β1-treated group, two-tailed Student’s test.Three independent experiments were carried out with three replicatesfor each experimental condition.

As a matter of fact, MRC5 treated with TGF-β1 expressed higher levels of these genes than control, while this increased expression was significantly reduced by SAHA (p ≤ 0.05; Fig. 6a,b).

Altogether, our *in vitro* analyses suggested that SAHA could inhibit TGF-β1 effect and could hinder lung fibrogenesis.

## Discussion

Though it is well known that BOS is due to inflammation-driven fibrotic processes, it represents a serious and unmet medical need and underlying molecular mechanisms of fibrogenesis are still poorly understood.

Recently, many studies have been highlighted the importance of lung resident MSC (LR-MSC) as key factors and predictors of BOS, which could promote fibrosis, and as an *in vitro* model to elucidate its molecular mechanisms.

The LR-MSC were firstly isolated from BALf of transplanted patients by Lama *et al*.^4^. They demonstrated that these cells are donor-derived, are able to differentiate *in vitro* to adipocytes, osteocytes and chondrocytes, and are characterized by the presence of mesenchymal and stem cell markers and by the absence of hematopoietic markers^4^.

In addition, they demonstrated that sex (male/female), type of LTx (single/bilateral) and indication for transplantation do not influence BOS onset or the number of mesenchymal CFUs isolated from BAL^4,6^. On the contrary, high numbers of MSC have been associated to BOS diagnosis, have been considered as predictor to future BOS onset^6^, and have been correlated with the presence of myofibroblasts in fibrotic lesions of lung allografts^7^.

As it has been demonstrated the MSC ability to differentiate in myofibroblasts, promoting lung fibrosis, and as epigenetic changes seem to be involved in lung fibrosis^8,10^ and in myofibroblast differentiation^22^, we hypothesized that the epigenetic determinants could contribute to BOS pathogenesis.

Interestingly, analyzing MSC isolated from BALf of BOS patients respect to those of stable LTRs, we found a deregulated expression of some epigenetic enzymes and several miRNAs. In particular, among them, we highlighted an upregulated expression of class I histone deacetylases. These data support the findings of earlier investigators that discover the key roles of class I HDACs in promotion of cardiac fibrosis (contrary to class II HDACs)^23^, and of several pan- or class I specific- HDAC inhibitors in reduction tissue fibrosis in heart, liver, skin and kidney and in suppression of fibroblast activation^23–25^.

Responsible of chromatin modifications associated with organ fibrosis are also DNA methyltransferase enzymes, DNMTs^12,26,27^, which are implicated in transcriptional silencing in concert with HDACs^28^.

Notably, our data are in line with previous findings of Sanders and coworkers^29^ that described an increase of *de novo* enzymes DNMT-3a and DNMT-3b in idiopathic pulmonary fibrosis (IPF) lungs, and of Dakhlallah *et al*. that reported DNMT1 expression increase in IPF lung tissues and lung fibroblast cell lines^30^, which is inversely correlated to FVC (forced expiratory vital capacity).

Moreover, several studies have revealed a differential DNA methylation pattern in IPF lung and control samples^29,31,32^, describing a hypermethylation of specific genes, such as CD90^33,34^ and PPAR-γ^26,35,36^ (whose silencing is DNMTs- and HDACs-dependent^37^), which contributes to myofibroblast differentiation. We found that MSC from BOS patients showed a reduced expression of these anti-fibrotic genes, together with an upregulation of fibrotic genes, confirming their profibrotic myofibroblastic phenotype.

In addition, we found a slight upregulation of some methyl-CpG binding proteins (MBD2 and MeCP2), which specifically recognize and bind to methylated DNA, and form a complex along with HDACs, thereby repressing gene expression^38^.

The importance of MeCP2 in myofibroblast differentiation and pulmonary fibrosis has been reported^39,40^: indeed MeCP2 control the expression of EZH2, PPARγ, and α-SMA, inducing myofibroblast differentiation and fibrosis^26,41^.

Based on these findings and our data, DNMTs/HDACs inhibitors along with MeCP2 could be considered as promising candidates for anti-fibrotic therapy.

Of particular importance is the contribution of miRNAs, widely recognized as key regulators of several pathophysiological processes, and as integrated part of a miRNA-epigenetics regulatory network^42^, also implicated in fibrotic diseases^27^.

Profibrotic phenotype of MSC from BOS patients was confirmed also by upregulation of pro-fibrotic mediators (such as miR-199 family, miR-142-3p, miR-146a, miR-155, miR-17/92 cluster, miR-18, miR-192, miR-21, miR-224, miR-23a/27a/24 cluster, miR-451, miR- 449, miR-7, miR-15/107 family, miR-365 miR-101, miR-144) and downregulation of anti-fibrotic miRNAs (miR-145, miR- 206, miR- 125b, let-7c)^19,43–45^.

Moreover, our data are in line with a very recent paper, in which it has been described, for the first time, the miRNA profile in murine LR-MSC after TGF-β1-induced myofibroblast differentiation^46^. Comparing with their results, we confirmed the upregulation of 88 miRNAs and the downregulation of miR-302a.

Interestingly, in MSC from BOS patients, the previously described upregulation of some epigenetic enzymes is accompanied by a downregulation of several miRNAs, whose expression is dependent on their enzyme activities^42^.

As a matter of fact, DNMTs also control the expression of some specific microRNAs, contributing to myofibroblast differentiation through silencing of certain gene transcription^27^. For example, MSC from BOS patients had lower expression levels of miR-124a, whose promoter has a large CpG island, which could be hypermethylated (and silenced), as in several tumors^47^. It has been described that there is a correlation between DNMT3b expression and the hypermethylation of miR-124a, and that 5-aza-2′-deoxycytidine (AZA) treatment induces the miRNA expression restoration^48–50^.

Other epigenetically silenced miRNAs, miR-338-3p^51^, miR-302b, miR-372, miR-520b, miR-523, miR-603, miR-136^50^, miR-516-3p^52^, miR-129^53^ are downregulated in MSC from BOS patients, and their expression can be restored after AZA and Trichostatin (TSA) treatments, suggesting that their silencing is due to DNMTs and HDACs^50^.

As the relationship between miRNAs and epigenetics is bidirectional, MSC from BOS patients have low levels of some miRNAs which negatively regulate epigenetic enzymes, which consequently result upregulated. In particular, we highlighted a downregulation of the cluster miR-302/367, key mediators of a several processes, including epigenetic regulation. Indeed, miR-302 cluster members target many epigenetic factors, such as MeCP1, MeCP2 and MBD2, leading to a global demethylation^54^.

Moreover, it is known that the expression of miR371 cluster is downregulated before that of the mir-302/367 cluster, during the stem cell differentiation^55^, and that miR-302/372 target MBD2^54^. Interestingly, MSC from BOS patients expressed miR-371 cluster at low levels, together to miR-302/367, while MeCP2 and MBD2 are overexpressed.

Among miRNAs which regulate epigenetic enzymes,miR-483-5p regulates MeCP2 levels and there is an inverse correlation of miR-438-5p and MeCP2 levels in developing human brains and fibroblasts from Beckwith-Wiedemann syndrome patients^56^, as revealed in MSC from BOS patients in our experimental setting.

In addition, we found in MSC from BOS patients a low expression levels of miR-1264 and an upregulation of DNMT1, in line with Boosani and coworkers that found that DNMT1 is targeted by this miRNA^57^.

In addition, in this study, using *in vitro* experiments with lung fibroblast cell line MRC5, we confirmed the central role of histone deacetylates in fibrogenesis, highlighting the possible use of histone deacetylase inhibitor to directly inhibit myofibroblast formation. Our data are in line with other groups that demonstrated that SAHA is able to attenuate the TGF-β1 effects on proliferation and differentiation^20,58,59^.

Altogether, the data here presented provide a potential lead into future mechanistic investigations regarding BOS pathogenesis, and, given the reversibility of epigenetic modifications, the understanding of these mechanisms may represent a promising novel therapeutic target for BOS.

The identified epigenetic profiles will thus pave the road for an epidrug screening and will provide preclinical evidence for epigenetic drugs as potential novel therapies to benefit BOS patients.

In addition, the identified molecular signature could be very useful to identify reliable biomarkers in BOS pathologies.

In summary, our study, for the first time, identifies epigenetic differences in MSC of BOS patients after lung transplantation, which may be, at least partially, responsible of their differentiation in myofibroblasts and may shed new lights on the understanding the molecular mechanisms of BOS onset. Nonetheless, there are some limitations to this exploratory study concerning small sample size and the use of immunosuppressive agents which could be influence gene expression, and future functional studies will be necessary in order to corroborate these findings and to unravel these questions and other concerns.

## Materials and Methods

### Patient and sample collection

The study was approved by the local Ethical Committee and by Institutional Research Review Boards of IRCCS ISMETT (IRRB/09/16) and Fondazione IRCCS Policlinico San Matteo Hospital (Project N° 20140003328. Date of approval: 01/09/2014).

All patients gave written informed consent to the collection. The investigation was conformed to the declaration of Helsinki, and all procedures were compliant with local and national legislations, regulations, and guidelines.

Study subjects were enrolled after informed consent, and were divided into 3 groups: controls consisting of stable lung recipients, BOS 0p patients with a diagnosis of potential BOS, and established BOS (grade 1–3) patients. For patient characteristics see Supplementary Table S1. The diagnosis of BOS were made by the criteria established by the International Society of Heart and Lung Transplantation^60^. The biopsy specimens and BALf were obtained during the post-transplant period for routine surveillance to rule out acute rejection or infection.

Study was conducted in a blinded manner. Indeed, sample were collected, deidentified, coded, and sent for the analyses. Assignment of subjects to groups, data recording and data analysis were blinded to the operators.

### MSC isolation and characterization

MSC were isolated from BALf of LTR, obtained following standard recommendations^61^, and cultured as previously described^62^.

Briefly, two million cells were seeded in high-glucose DMEM Gibco, USA) with 10% fetal calf serum, and 100 units ml-1 penicillin/streptomycin solution. Single foci of MCs formed between 7–28 days, and representing the only remained cells, were isolated and collected. Afterwards, they were characterized using a panel of antibodies specific for mesenchimal cells and spread in new culture dishes. Surface phenotypes of all isolated MCs at first passage were evaluated as previously described by Lama and collegues^4^, using fluorochrome-labeled antibodies (CD44-FITC, CD105-FITC, CD90-PE, CD34-ECD and CD45-APC750 [Instrumentation Laboratory; Milan, Italy]) by flow cytometry (Navios™ [Beckman Coulter Inc.; CA, USA] Flow cytometer) and data analyzed using Kaluza® software (Beckman Coulter Inc.).

In addition, MSC were characterized for FOXF1, a lung mesenchymal marker, by quantitative RT-PCR (qRT-PCR).

For the experiments, MSC, between the second and sixth passage after isolation, were used.

### RNA extraction

Total RNA was isolated from MSC by using the miRNeasy mini kit (Qiagen, CA), or from Formalin-Fixed, Paraffin-Embedded (FFPE) slides by using RecoverAll Total Nucleic Acid Isolation Kit for FFPE (Life Technologies, Thermo Fisher Scientific, Waltham, MA, USA), according to the manufacturers’ instructions.

For each sample, after RNA extraction, we verified RNA integrity and genomic DNA (gDNA) contamination, using Agilent BioAnalyzer (Agilent Technologies Inc., Waldbroon, Germany).

### Analysis of epigenetic genes

The cDNA for each RNA sample was obtained using RT² First Strand kit (SABiosciences Corporation, Qiagen, Frederick, MD, USA), according to the manufacturer’s instructions. The expression levels of epigenetic enzymes were analyzed using RT² Profiler™ PCR Array - Human Epigenetics chromatin modification enzymes (PAHS-085ZC; SABiosciences Corporation, Qiagen), following the manufacturer’s instructions. An Applied Biosystems StepOne Plus PCR System (Life Technologies, Thermo Fisher Scientific) was used for measurements. The PCR array data were analyzed by RT^2^ Profiler PCR Data Analysis software (SABiosciences Corporation; http://sabiosciences.com/pcrarraydataanalysis.php). Relative quantification for each gene was assessed by 2^−ΔΔCT^ calculation for each mRNA. All test samples were run in duplicate and template-negative reactions served as controls. The array data were validated using singles assays for TaqMan qRT-PCR (Life Technologies, Thermo Fisher Scientific) for DNMT1 (Hs00945875_m1), DNMT3A (Hs01027166_m1), HAT1 (Hs00186320_m1), HDAC1 (Hs02621185_s1) and HDAC4 (Hs01041638_m1). EZH2 (Hs01016789_m1) and MECP2 (Hs00172845_m1)were also analyzed by single assays. GAPDH (Hs02758991_g1) was used as a reference gene for the relative quantification, assessed by 2^−ΔΔCT^ method for each mRNA. GO Gene enrichment analysis was performed by the Gene Ontology (GO) database (http://geneontology.org/page/go-enrichment-analysis) using PANTHER (http://pantherdb.org/), for finding statistically overrepresented functional groups within the upregulated genes in BOS patients respect to stable LTRs. The number of genes for each category were also listed (p ≤ 0.05).

### Gene expression analysis

Reverse transcription was performed using the High-Capacity cDNA Reverse Transcription Kit (Life Technologies, Thermo Fisher Scientific) according to the manufacturer’s instructions. Gene expression was quantified by qRT-PCR using StepOne Plus Real-Time instrument using TaqMan or Sybr Green methods. TaqMan gene assays (Life Technologies, Thermo Fisher Scientific) for CD90 (Hs00174816_m1), PPARγ (Hs00234592_m1), FN1 (Hs01549976_m1), TGF-β1 (Hs00998133_m1)and GAPDH (Hs02758991_g1) were used. The sequences of forward and reverse primers for Sybr Green method were: COL1A1 Forward 5′-CACCCTTAGCACCAACAG-3′ and Reverse 5′-GACACAGAGGTTTCAGTGG-3′; CTGF Forward 5′-AGGAGTGGGTGTGTGACGA-3′ and Reverse 5′-CCAGGCAGTTGGCTCTAATC-3′; GAPDH Forward 5′-ACCAGGAAATGAGCTTGACAAA-3′ and Reverse 5′-CGAGATCCCTCCAAAATCAA-3′.

GAPDH was used as a reference gene for the relative quantification, assessed by 2^−ΔΔCT^ calculation for each mRNA. The samples were run in duplicate and template-negative reactions served as controls.

### miRNAs profiling

MiRNA profiling was performed using TaqMan Array Human MicroRNA panels A and B (Life Technologies, Thermo Fisher Scientific) to analyze 754 human miRNAs. Reverse transcription and pre-amplification were performed following the manufacturer’s instructions (Life Technologies, Thermo Fisher Scientific). Real-time RT-PCR was performed with the Applied Biosystems StepOne Plus PCR system. For each miRNA, the expression level was determined by the equation 2^−ΔΔCT^. Data Assist software (v 3.01) (Life Technologies, Thermo Fisher Scientific) was used to process the array data. Results were validated using single assays for TaqMan qRT-PCR (Life Technologies, Thermo Fisher Scientific) for miR-106a (002169), miR-124 (001182), miR-146a (000468), miR-18b (002217) and miR-372 (000560). U6 snRNA (001973) was used as control to normalize data.

Cluster 3.0/TreeView programs were used for hierarchical clustering analysis of miRNA expression to arrange the samples into groups based on their expression levels, as described by Chiang *et al*., 2011^63^. Prior to hierarchical clustering data was log_2_ transformed and expression values on all arrays were translated to the same mean. Spearman rank correlation coefficient was used for calculation of distances between miRNAs. The hierarchical clustering tree was inferred from the distances using average linkage method.

### MRC5: cell culture and treatments

MRC5 cells, a cell line of human lung fibroblasts (BS CL 68, IZSLER-BVR, Brescia, Italy), were grown in Eagle’s minimal essential medium (MEM, Gibco, USA) supplemented with 2 mM L-glutamine, 100 U/ml penicillin, 100 mg/ml streptomycin and 10% fetal bovine serum.

MRC5 cells were incubated in a humidified incubator at 37 °C and 5% CO to 60–80% confluence, and growth arrested for 24 h in DMEM with 0.01% FBS before adding 10 ng/ml rhTGF-β1 (AF-100-21C, Peprotech, USA) or 5 µM SAHA (CAY-10009929-100, Cayman Chemical, USA) for 24 h.

SAHA (suberoylanilide hydroxamic acid, Vorinostat, Zolinza) is a small-moleculeinhibitor of class I and class II HDACwith wide spectrum of epigenetic activities^64,65^. It has been demonstrated that SAHA inhibition of HDAC promotes cell cyclearrest, differentiation, and apoptosis of cancer cells^64,66,67^. It was the first HDAC inhibitor approved by the FDA for cancer therapy.

### Cell cycle assays

After treatment, MRC5 cells were analyzed by flow cytometry. Briefly, cells were harvested by trypsinization 24 h after treatment, washed in ice-cold PBS, fixed in cold 70% ethanol to a final concentration of 10^6^ cells/ml for 1 h at 4 °C. The ethanol-fixed cells were centrifuged at 300× for 10 min to remove ethanol and the pellet was stained in the dark with a solution containing 50 μg/ml propidium iodide and 50 μg/ml RNase (Sigma Chemical Co., St Louis, MO, USA) for 30 min at room temperature. The stained cells were analyzed for cell cycle using a FacsCantoII (BD Biosciences, San Diego, CA, USA), equipped with a FACSDiva 8.0.1. The percentage of cells in G0/G1, S, and G2/M phases was analyzed by ModFit LT (Verity Software House, Topsham, ME) and expressed as relative change compared to untreated cells.

### Apoptosis analysis

Apoptosis was analyzed by flowcytometry, using Annexin V (Annexin V-FITC Apoptosis Detection Kit I; BD Biosciences, Oxford, UK) according to the manufacturer’s instructions. The samples were analyzed using FacsAriaII sorter (BD Biosciences, San Diego, CA, USA). For each sample, 20,000 events were acquired. The data was analyzed using FACSDiva 8.0.1 (BD).

### Cell proliferation assays

For cell counting studies, untreated and treated MRC5 cells were counted taking advantage of a hemocytometer after trypan blue staining.

Cell proliferation was also assessed by xCELLigence RTCA MP System (Roche, Germany) that monitors cellular events in real time by measuring electrical impedance across interdigitated gold microelectrodes integrated on the bottom of culture plates. The impedance measurement provides quantitative information about the biological status of the cells, including cell number, viability and morphology^68^. Cell-sensor impedance is expressed as an arbitrary unit called Cell Index (CI). To determine the Cell Index, 6,000 cells were seeded into 100 μl of standard medium in 96× microtiter plates (E-Plate-Roche, Germany). Background impedance was determined using 100 μl of standard medium. Cell attachment, spreading and proliferation were monitored every 30 min using the xCELLigence system. Cell proliferation was monitored for 72 hr. Experimental results were performed using RTCA Software 1.2 that calculated the population doubling by fitting the curve to an exponential equation.

### Cell migration assays

MRC5 were plated densely in a six-well tissue culture plate. A scratch was made using a P200 (horizontally) or a P1000 (vertically) pipette tips. The cells were then washed with PBS three times to remove dislodged cells, and further cultured in DMEM with TGF-β1 and/or SAHA. Cells were left to migrate for 17 h at 37 °C under a 5% CO_2_. Wound closure was monitored by collecting digitized images at 0 and 17 h after the scratch was performed, with an inverted microscope (EVOS, Thermo Fisher Scientific). The digitized images were then analyzed using ImageJ software as previously described^69^. Using the ImageJ program, the size of the denuded area was determined at each time point from the digital images. The gap width of scratch closure was measured compared with the initial gap size at 0 h, and expressed as percentage change.

### Statistical analysis

QRT-PCR data analyses were conducted using tools from RQ-manager (v1.2, Life Technologies, Thermo Fisher Scientific), Microsoft Excel and Prism GraphPad V5.0d software (GraphPad Software, CA). Results were expressed as Mean (SD). Statistical significance of observed differences among different groups was calculated using a two-tailed unpaired Student’s t test. P-values ≤ 0.05 were considered to be statistically significant. In the figures, * and ** indicate statistical significance at p ≤ 0.05 and 0.01, respectively, compared to control group; or # and ## indicate statistical significance at p ≤ 0.05 and 0.01, respectively, compared to TGF-β1-treated group. Heat maps were constructed using Gene Cluster 3.0/TreeView programs (http://taxonomy.zoology.gla.ac.uk/rod/treeview.html).

## Electronic supplementary material

Supplementary Figures

Supplementary Tables
