# Supplementary material for: Lung resident mesenchymal cells isolated from patients with the Bronchiolitis Obliterans Syndrome display a deregulated epigenetic profile
Source: Sci Rep. 2018 Jul 24;8:11167. doi: 10.1038/s41598-018-29504-5 (PMC6057887; doi:10.1038/s41598-018-29504-5)
Supplement: Supplementary file 1 — Supplementary Figures [file 41598_2018_29504_MOESM1_ESM.pdf]

Lung resident mesenchymal cells isolated from patients with the Bronchiolitis Obliterans Syndrome display a deregulated epigenetic profile.

Serena Vella[1,2,*], Pier Giulio Conaldi[1,3], Emanuela Cova[4], Federica Meloni[4], Rosa Liotta[5], Salvatore Cuzzocrea[6], Lavinia Martino[7], Alessandro Bertani[7], Angelo Luca[8], Patrizio Vitulo[7]

[1]Department of Laboratory Medicine and Advanced Biotechnologies, IRCCS-ISMETT (Istituto Mediterraneo per i Trapianti e Terapie ad Alta Specializzazione), Palermo, Italy.
[2]Current address: Anemocyte S.r.l., Gerenzano, Italy.
[3] Fondazione Ri.MED, Palermo, Italy.
[4]Department of Respiratory Diseases, IRCCS San Matteo Foundation and University of Pavia, Pavia, Italy.
[5]Department of Diagnostic and Therapeutic Services, Pathology Service, IRCCS-ISMETT (Istituto Mediterraneo per i Trapianti e Terapie ad Alta Specializzazione), Palermo, Italy.
[6]Department of Chemical, Biological, Pharmaceutical and Environmental Sciences, University of Messina, Messina, Italy.
[7]Department for the Treatment and Study of Cardiothoracic Diseases and Cardiothoracic Transplantation, IRCCS-ISMETT (Istituto Mediterraneo per i Trapianti e Terapie ad Alta Specializzazione), Palermo, Italy.
[8]Department of Diagnostic and Therapeutic Services, Radiology Service, IRCCS-ISMETT (Istituto Mediterraneo per i Trapianti e Terapie ad Alta Specializzazione), Palermo, Italy.

*** Corresponding author:**
Serena Vella
Email: vellaserena@gmail.com

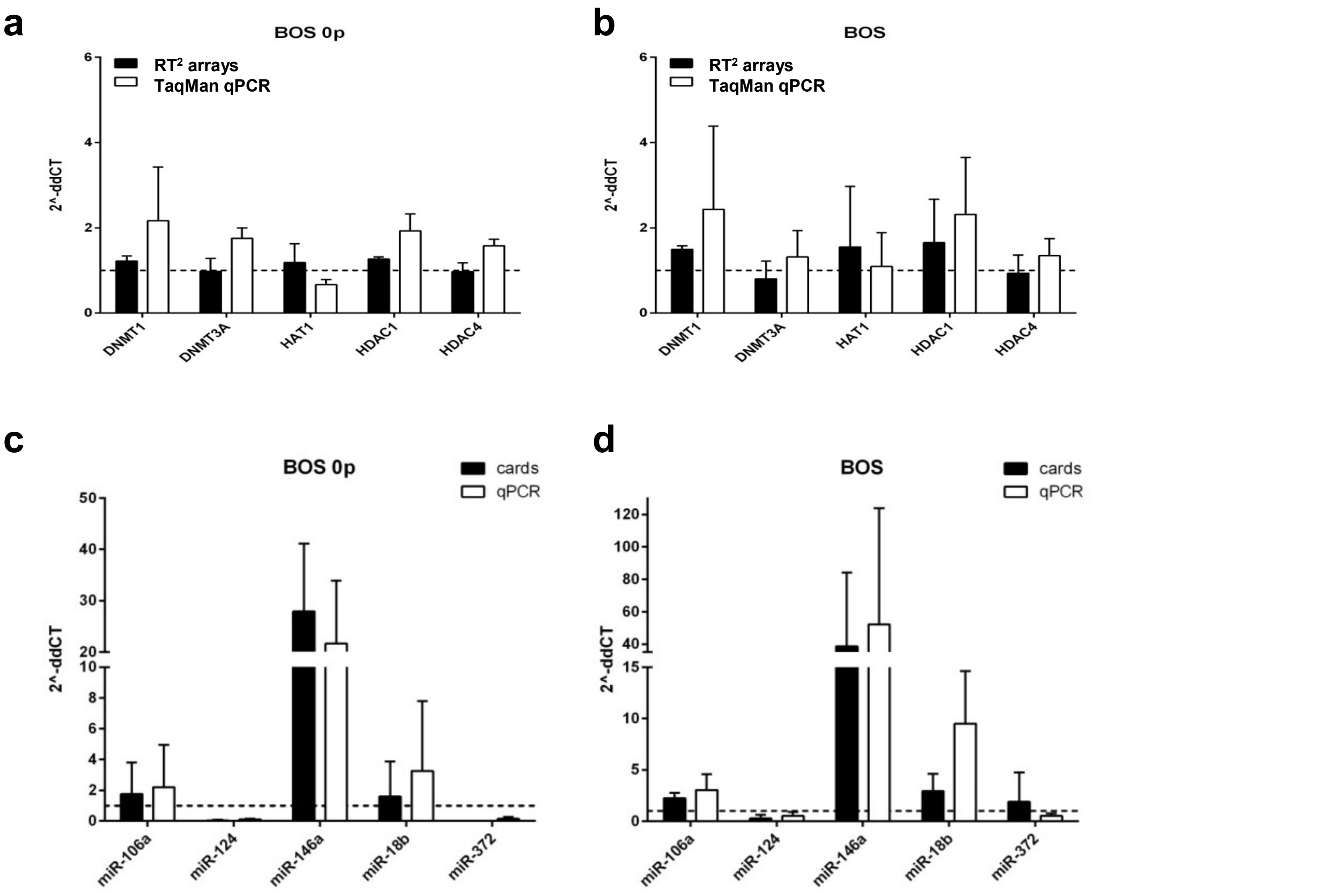

Supplementary Figure S1.*Validation of RT^2 array-based gene expression profile by qRT-PCR.*
Values represent fold change in transcript copy number relative to control GAPDH. Mean (SD) expression of the DNMT1, DNMT3A, HAT1 and HDAC4, as measured by RT^2 arrays and TaqMan qRT-PCR in MSC from BALf of BOS 0p *vs* stable LTRs, **a)**, and BOS *vs* stable LTRs, **b)**.
Validation of array-based gene expression profile by qRT-PCR. Values represent fold change in transcript copy number relative to control U6. Mean (±SD) expression of the miR-106a, miR-124, miR-146a, miR-18b and miR-372, as measured by TLDA array and qRT-PCR in MSC from BALf of BOS 0p *vs* stable LTRs, **c)**, and BOS *vs* stable LTRs, **d)**.

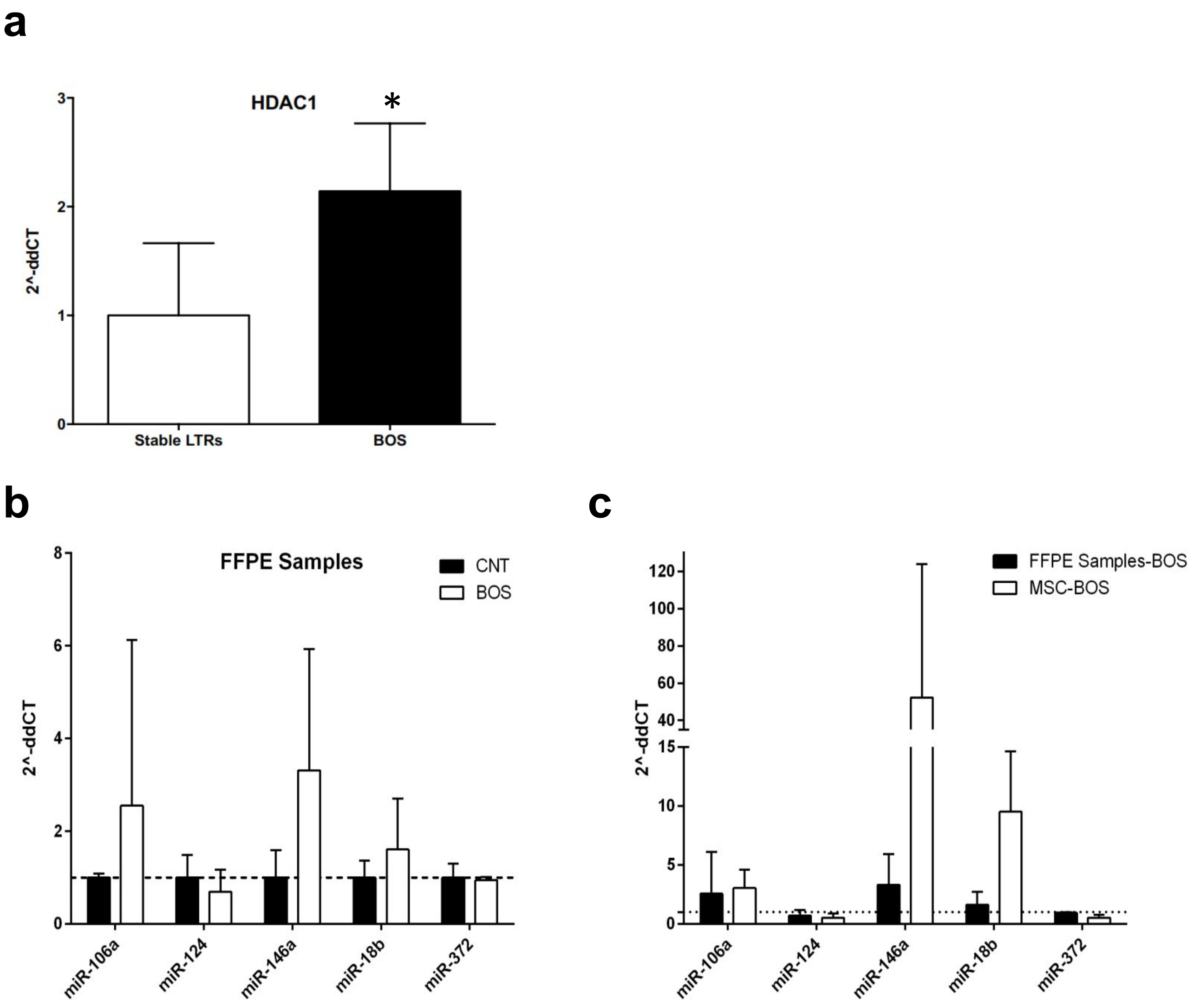

Supplementary Figure S2.*Epigenetic changes in human BOS lung biopsies.*

a) QRT-PCR results of HDAC1 in lung tissues from BOS patients (n=4) and stable LTRs (n=4). Data are expressed as Mean (±SD). * $p \leq 0.05$, two-tailed Student's test.

b) MiRNA expression analysis in FFPE samples of BOS patients (n=4) and stable LTRs (n=4). Values represent fold change in transcript copy number relative to control U6, analysed by qRT-PCR. Data are expressed as Mean (±SD).

c) Mean (±SD) expression of some miRNAs, measured by qRT-PCR, in MSC from BALf of BOS (n=3) and in FFPE BOS samples (n=4).

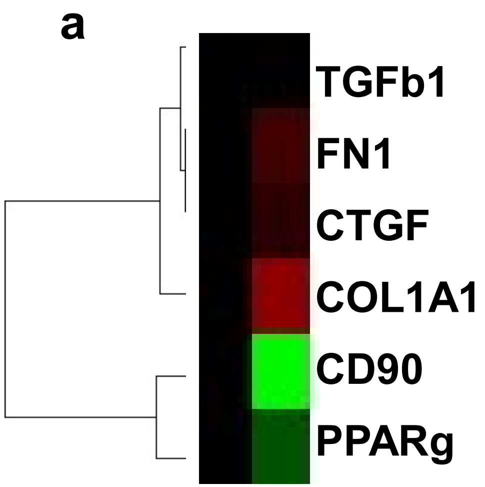

Supplementary Figure S3.*Gene expression changes in MSC from BOS patients.*

a) Non-supervised hierarchical clustering based on the gene expression levels of pro-fibrotic genes (TGF-β1, FN, Col1A1 and CTGF) and anti-fibrotic genes (PPARγ and CD90) in MSC from BALf of stable LTRs and BOS (n=5 and n=3, respectively). The dendrogram shows the relationships among gene expression patterns: ‘‘red’’ indicates high relative expression, ‘‘black’’ no change, and "green" low relative expression.

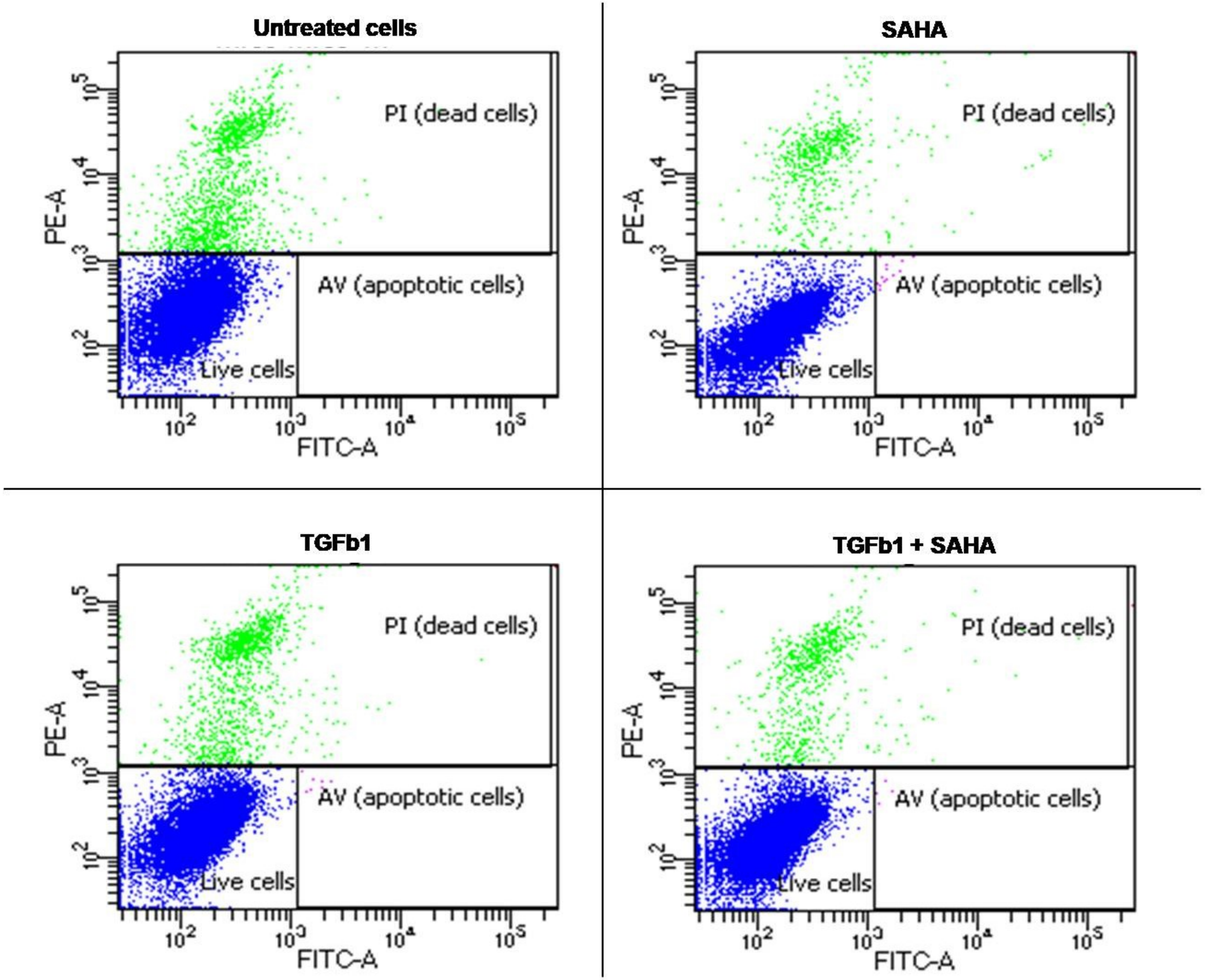

Supplementary Figure S4.*Annexin V/PI staining.*

Diagrams of FITC-Annexin V/PI flowcytometry of treated and untreated MRC5. The lower left quadrants of eachpanel show the viable cells (negative for PI and FITC-Annexin V binding). The upper quadrants contain thenecrotic cells, positive for PI uptake. The lower right quadrants represent the apoptotic cells, FITC-Annexin V positive and PI negative. One representative experiment for each experimental condition is shown. The averaged percentageof viable, necrotic and apoptotic cells are represented in figure 4d.

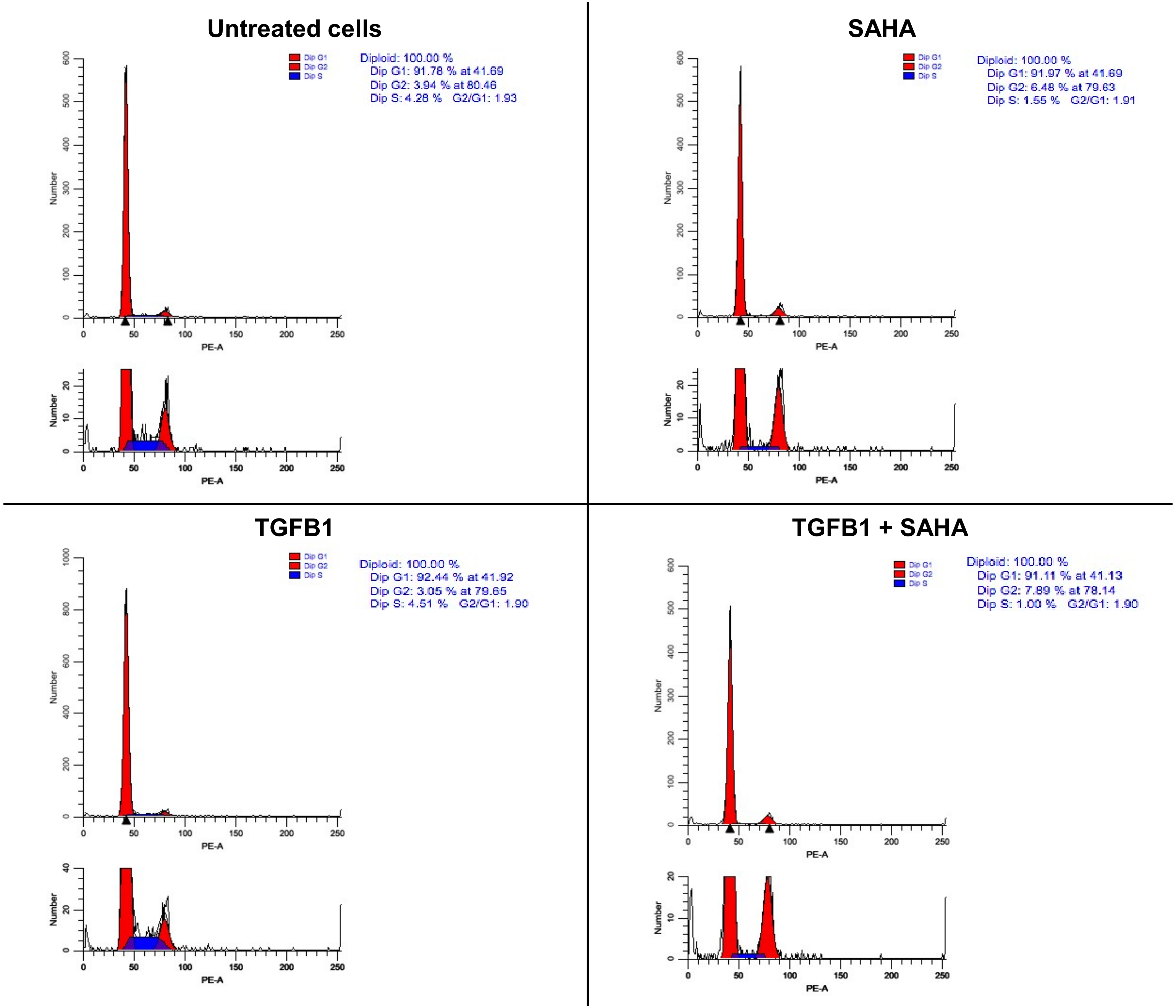

Supplementary Figure S5.*Cell cycle analysis with PI staining.*

Cell cycle phase distributions of MRC5 treated with or without SAHA and/or TGF-β1, analyzed by ModFit LT software. The data are representative as a mean of 3 experiments with similar results, which are shown in the figure 4e.
