# Supplementary material for: Lung resident mesenchymal cells isolated from patients with the Bronchiolitis Obliterans Syndrome display a deregulated epigenetic profile
Source: Sci Rep. 2018 Jul 24;8:11167. doi: 10.1038/s41598-018-29504-5 (PMC6057887; doi:10.1038/s41598-018-29504-5)
Supplement: Supplementary file 2 — Supplementary Tables [file 41598_2018_29504_MOESM2_ESM.docx]

***Lung resident mesenchymal cells isolated from patients with the Bronchiolitis Obliterans Syndrome display a deregulated epigenetic profile.***

Serena Vella^1,2,*^, Pier Giulio Conaldi^1,3^, Emanuela Cova^4^, Federica Meloni^4^, Rosa Liotta^5^, Salvatore Cuzzocrea^6^, Lavinia Martino^7^, Alessandro Bertani^7^, Angelo Luca^8^, Patrizio Vitulo^7^

^1^Department of Laboratory Medicine and Advanced Biotechnologies, IRCCS-ISMETT (Istituto Mediterraneo per i Trapianti e Terapie ad Alta Specializzazione), Palermo, Italy.

^2^Current address: Anemocyte S.r.l., Gerenzano, Italy.

^3^ Fondazione Ri.MED, Palermo, Italy.

^4^Department of Respiratory Diseases, IRCCS San Matteo Foundation and University of Pavia, Pavia, Italy.

^5^Department of Diagnostic and Therapeutic Services, Pathology Service, IRCCS-ISMETT (Istituto Mediterraneo per i Trapianti e Terapie ad Alta Specializzazione), Palermo, Italy.

^6^Department of Chemical, Biological, Pharmaceutical and Environmental Sciences, University of Messina, Messina, Italy.

^7^Department for the Treatment and Study of Cardiothoracic Diseases and Cardiothoracic Transplantation, IRCCS-ISMETT (Istituto Mediterraneo per i Trapianti e Terapie ad Alta Specializzazione), Palermo, Italy.

^8^Department of Diagnostic and Therapeutic Services, Radiology Service, IRCCS-ISMETT (Istituto Mediterraneo per i Trapianti e Terapie ad Alta Specializzazione), Palermo, Italy.

*** Corresponding author:**

Serena Vella

Email: vellaserena@gmail.com

**Supplementary Table S1:** Clinical characteristics of patients.

| **MSC from BALf** | Sample | Age | Gender | Diagnosis | Type of LTx | BOS |
| --- | --- | --- | --- | --- | --- | --- |
| **Stable LTRs** | 4069 | 44 | M | NSIP | Single | No |
|  | 4175 | 65 | M | UIP | Single | No |
|  | 4205 | 44 | F | NSIP | Single | No |
|  | 3887 | 57 | M | CPFE | Bilateral | No |
|  | 3911 | 71 | M | E | Bilateral | No |
| Median (range) |  | 56.2 (12.19) |  |  |  |  |
| **BOS** | 4352 | 66 | F | NSIP | Single | Yes  (BOS0p) |
|  | 3780 | 38 | F | NSIP | Single | Yes  (BOS0p) |
|  | 3649 | 58 | M | RF | Single | Yes  (BOS1) |
|  | 4000 | 38 | M | CF | Bilateral | Yes  (BOS1) |
|  | 3995 | 38 | M | PPH | Bilateral | Yes  (BOS3) |
| Median (range) |  | 47.6 (13.45) |  |  |  |  |

| **FFPE lung biopsy** | Sample | Age | Gender | Diagnosis | Type of LTx | BOS |
| --- | --- | --- | --- | --- | --- | --- |
| **Stable LTRs** | 40959 | 59 | M | IPF | Bilateral | No |
|  | 30898 | 59 | M | IPF | Single | No |
|  | 25174 | 27 | M | LCH | Bilateral | No |
|  | 33526 | 43 | M | IPF | Bilateral | No |
| Average (SD) |  | 47 (15.32) |  |  |  |  |
| **BOS** | 34368 | 52 | M | CF | Bilateral | Yes  (C1) |
|  | 30584 | 66 | M | IPF | Bilateral | Yes (A0B0C1) |
|  | 36465 | 46 | M | IPF | Bilateral | Yes (A3B1C1) |
|  | 32011 | 47 | F | IPF | Bilateral | Yes (A1B2C1) |
| Median (range) |  | 52.75 (9.21) |  |  |  |  |

**NSIP**: Non-specific interstitial pneumonia

**UIP**: Usual interstitial pneumonia

**CPFE**: Combined pulmonary fibrosis and emphysema

**E**: Emphysema

**RF**: Radiation Fibrosis

**PPH**: Primary pulmonary hypertension

**CF**: Cystic fibrosis

**LCH**: Langerhans cell histiocytosis

**Supplementary Table S2.** Genes over-expressed in MSC from BOS 0p patients vs Control Group (fold change ≥ 1.5).

| Gene | Fold Change | p-value |
| --- | --- | --- |
| RPS6KA5 | 2.9233 | 0.023325 |
| HDAC2 | 1.7719 | 0.000605 |
| SETDB2 | 1.7554 | 0.541659 |
| DZIP3 | 1.7383 | 0.016134 |
| PRMT6 | 1.723 | 0.079819 |
| RNF2 | 1.7164 | 0.012637 |
| MECP2 | 1.7077 | 0.841718 |
| HDAC1 | 1.7061 | 0.087215 |
| HDAC3 | 1.5958 | 0.001826 |
| NCOA1 | 1.5722 | 0.07958 |
| HDAC5 | 1.5676 | 0.107408 |
| RPS6KA3 | 1.5614 | 0.007886 |
| MBD2 | 1.5332 | 0.050273 |
| CIITA | 1.5122 | 0.652307 |
| UBE2A | 1.5078 | 0.027294 |

**Supplementary Table S3.** Genes down-regulated in MSC from BOS 0p patients vs Control Group (fold change ≤ 0.5).

| Gene | Fold Change | p-value |
| --- | --- | --- |
| ESCO2 | 0.4227 | 0.131933 |
| AURKB | 0.4264 | 0.121206 |

**Supplementary Table S4.** Genes over-expressed in MSC from BOS patients vs Control Group (fold change ≥ 1.5).

| Gene | Fold Change | p-value |
| --- | --- | --- |
| RPS6KA5 | 3.281 | 0.017646 |
| CIITA | 2.7344 | 0.001783 |
| DZIP3 | 2.2063 | 0.000488 |
| SETDB2 | 2.0777 | 0.161377 |
| MECP2 | 2.068 | 0.295311 |
| HDAC2 | 2.0453 | 0.000845 |
| PRMT8 | 1.9715 | 0.031952 |
| HDAC1 | 1.9667 | 0.012195 |
| UBE2A | 1.8869 | 0.003514 |
| RNF2 | 1.8239 | 0.012785 |
| PRMT3 | 1.7812 | 0.000519 |
| UBE2B | 1.727 | 0.011008 |
| USP16 | 1.7247 | 0.007111 |
| NEK6 | 1.6612 | 0.128402 |
| EZH2 | 1.6297 | 0.306982 |
| MYSM1 | 1.6281 | 0.002822 |
| HDAC3 | 1.6235 | 0.000007 |
| DNMT3B | 1.5827 | 0.020132 |
| DNMT1 | 1.5812 | 0.403073 |
| KAT6A | 1.5705 | 0.060173 |
| HDAC8 | 1.5428 | 0.008882 |
| KAT6B | 1.5278 | 0.027449 |
| NCOA6 | 1.5105 | 0.006834 |

**Supplementary Table S5.** GO molecular function of genes over-expressed in MSC from BOS patients vs Control Group (fold change ≥ 1.5).

| GO molecular function | Genes |
| --- | --- |
| DNA-methyltransferase activity (GO:0009008) | DNMT3B, DNMT1 |
| DNA (cytosine-5-)-methyltransferase activity, acting on CpG substrates (GO:0051718) | DNMT3B, DNMT1 |
| DNA (cytosine-5-)-methyltransferase activity (GO:0003886) | DNMT3B, DNMT1 |
| unmethylated CpG binding (GO:0045322) | HDAC1, HDAC2 , HDAC3 |
| NF-kappaB binding (GO:0051059) | DNMT3B, DNMT1, MBD2 |
| structure-specific DNA binding (GO:0043566) | MBD2, HDAC1, HDAC2 , EZH2 |
| protein methyltransferase activity (GO:0008276) | HDAC1, HDAC2, HDAC3, HDAC8 |
| protein deacetylase activity (GO:0033558) | HDAC1, HDAC2, HDAC3, HDAC8 |
| N-methyltransferase activity (GO:0008170) | SETDB2, PRMT3, EZH2, PRMT8 |
| NAD-dependent protein deacetylase activity (GO:0034979) | HDAC1, HDAC2, HDAC3, HDAC8 |
| NAD-dependent histone deacetylase activity (H3-K14 specific) (GO:0032041) | HDAC1, HDAC2, HDAC3, HDAC8 |
| NAD-dependent histone deacetylase activity (GO:0017136) | HDAC1, HDAC2, HDAC3, HDAC8 |
| hydrolase activity, acting on carbon-nitrogen (but not peptide) bonds, in linear amides (GO:0016811) | HDAC1, HDAC2, HDAC3, HDAC8 |
| hydrolase activity, acting on carbon-nitrogen (but not peptide) bonds (GO:0016810) | HDAC1, HDAC2, HDAC3, HDAC8 |
| histone methyltransferase activity (GO:0042054) | SETDB2, PRMT3, EZH2, PRMT8 |
| histone deacetylase activity (H3-K14 specific) (GO:0031078) | HDAC1, HDAC2, HDAC3, HDAC8 |
| histone deacetylase activity (GO:0004407) | HDAC1, HDAC2, HDAC3, HDAC8 |
| deacetylase activity (GO:0019213) | HDAC1, HDAC2, HDAC3, HDAC8 |
| chromatin DNA binding (GO:0031490) | HDAC1, HDAC2, MBD2, EZH2 |
| transcription coactivator activity (GO:0003713) | NCOA6, USP16, CIITA, KAT6A, MYSM1 |
| histone deacetylase binding (GO:0042826) | DNMT3B, MBD2, HDAC3, HDAC1, DNMT1 |
| transferase activity, transferring one-carbon groups (GO:0016741) | DNMT3B, DNMT1, SETDB2, PRMT3, EZH2, PRMT8 |
| S-adenosylmethionine-dependent methyltransferase activity (GO:0008757) | DNMT3B, DNMT1, SETDB2, PRMT3, EZH2, PRMT8 |
| methyltransferase activity (GO:0008168) | DNMT3B, DNMT1, SETDB2, PRMT3, EZH2, PRMT8 |
| transcription factor binding (GO:0008134) | MBD2, NCOA6 , KAT6B, HDAC3, HDAC1, CIITA, HDAC8, KAT6A, HDAC2 |
| transcription factor activity, transcription factor binding (GO:0000989) | DNMT3B, MBD2, NCOA6, USP16, HDAC3, HDAC1, CIITA, KAT6A, MYSM1 |
| transcription factor activity, protein binding (GO:0000988) | DNMT3B, MBD2, NCOA6, USP16, HDAC3, HDAC1, CIITA, KAT6A, MYSM1 |
| transcription cofactor activity (GO:0003712) | DNMT3B, MBD2, NCOA6, USP16, HDAC3, HDAC1, CIITA, KAT6A, MYSM1 |
| chromatin binding (GO:0003682) | DNMT3B, MBD2, NCOA6, HDAC3, HDAC1, DNMT1, RNF2, HDAC2, EZH2 |
| enzyme binding (GO:0019899) | UBE2A, DNMT3B, MBD2, DZIP3, NCOA6, NEK6, UBE2B, HDAC3, HDAC1, DNMT1, HDAC2 |
| macromolecular complex binding (GO:0044877) | DNMT3B, MBD2, NCOA6, KAT6B, HDAC3, HDAC1, CIITA, RNF2, HDAC2, MYSM1, EZH2 |
| transferase activity (GO:0016740) | UBE2A, DNMT3B, MBD2, DZIP3, NEK6, UBE2B, KAT6B, DNMT1, CIITA, SETDB2, RNF2, PRMT3, KAT6A, EZH2, RPS6KA5, PRMT8 |
| catalytic activity (GO:0003824) | UBE2A, DNMT3B, DZIP3, USP16, NEK6, UBE2B, KAT6B, DNMT1, HDAC3, HDAC1, CIITA, SETDB2, RNF2, PRMT3, HDAC8, KAT6A, HDAC2, MYSM1, EZH2, RPS6KA5, PRMT8 |
| protein binding (GO:0005515) | UBE2A, DNMT3B, DZIP3, USP16, NEK6, UBE2B, KAT6B, DNMT1, HDAC3, HDAC1, CIITA, SETDB2, RNF2, PRMT3, HDAC8, KAT6A, HDAC2, MYSM1, EZH2, RPS6KA5, PRMT8, MBD2, NCOA6 |

**Supplementary Table S6.** Fold regulation and p-values of enzymes with histone deacetylase or methyltransferase activity in MSC from BOS 0p patients vs Control Group.

| Functional groups | Gene | Fold Regulation (vs CNT) | pvalue |
| --- | --- | --- | --- |
| Histone deacetylase activity | HDAC1 | 1.9667 | 0.012195 |
|  | HDAC3 | 1.6235 | 0.000007 |
|  | HDAC8 | 1.5428 | 0.008882 |
|  | HDAC2 | 2.0453 | 0.000845 |
| Methyltransferase activity | DNMT3B | 1.5827 | 0.020132 |
|  | DNMT1 | 1.5812 | 0.403073 |
|  | SETDB2 | 2.0777 | 0.161377 |
|  | PRMT3 | 1.7812 | 0.000519 |
|  | EZH2 | 1.62961 | 0.306982 |
|  | PRMT8 | 1.9715 | 0.031952 |

**Supplementary Table S7.** Significant deregulated miRNAs in MSC from BOS patients (compared to those from stable LTRs) (p ≤ 0.05).

| miRNA | P value | Mean1- stable LTRs | Mean2- BOS | Difference | SE of difference | t ratio | df |
| --- | --- | --- | --- | --- | --- | --- | --- |
| miR-98 | 0.011517 | 1 | 570118 | -570117 | 158849 | 3.58905 | 6 |
| let-7b# | 0.038835 | 1 | 12819.9 | -12818.9 | 4866.14 | 2.63431 | 6 |
| let-7a# | 0.039793 | 1 | 4753.21 | -4752.21 | 1816.51 | 2.61612 | 6 |
| miR-500 | 0.036645 | 1 | 70.872 | -69.872 | 26.0934 | 2.67777 | 6 |
| miR-450b-5p | 0.03461 | 1 | 36.5338 | -35.5338 | 13.0604 | 2.72072 | 6 |
| miR-624 | 0.034554 | 1 | 31.0294 | -30.0294 | 11.0324 | 2.72193 | 6 |
| miR-16-1# | 0.027992 | 1 | 24.6085 | -23.6085 | 8.19232 | 2.88178 | 6 |
| miR-26a-2# | 0.035177 | 1 | 21.6069 | -20.6069 | 7.6083 | 2.70847 | 6 |
| miR-369-5p | 0.041318 | 1 | 19.1689 | -18.1689 | 7.02021 | 2.58809 | 6 |
| miR-369-3p | 0.048489 | 1 | 14.9525 | -13.9525 | 5.64989 | 2.46952 | 6 |
| miR-126# | 0.038298 | 1 | 14.2463 | -13.2463 | 5.00859 | 2.64473 | 6 |
| miR-7# | 0.044091 | 1 | 13.7334 | -12.7334 | 5.01347 | 2.53983 | 6 |
| miR-576-3p | 0.025429 | 1 | 13.2402 | -12.2402 | 4.14142 | 2.95556 | 6 |
| miR-590-3P | 0.02867 | 1 | 12.0835 | -11.0835 | 3.87061 | 2.86351 | 6 |
| miR-656 | 0.034865 | 1 | 11.6355 | -10.6355 | 3.91704 | 2.71518 | 6 |
| miR-301b | 0.044478 | 1 | 11.0474 | -10.0474 | 3.96603 | 2.53336 | 6 |
| miR-579 | 0.019312 | 1 | 10.4067 | -9.40669 | 2.96713 | 3.17029 | 6 |
| miR-29b | 0.009048 | 1 | 9.68861 | -8.68861 | 2.29108 | 3.79236 | 6 |
| miR-199b | 0.008477 | 1 | 7.97715 | -6.97715 | 1.81308 | 3.84824 | 6 |
| miR-101 | 0.048433 | 1 | 7.70172 | -6.70172 | 2.71283 | 2.47038 | 6 |
| miR-542-3p | 0.043178 | 1 | 7.52793 | -6.52793 | 2.5546 | 2.55536 | 6 |
| miR-455 | 0.026115 | 1 | 7.43916 | -6.43916 | 2.19389 | 2.93504 | 6 |
| miR-18a | 0.025721 | 1 | 7.34824 | -6.34824 | 2.15432 | 2.94674 | 6 |
| miR-10b | 0.007149 | 1 | 7.10499 | -6.10499 | 1.52767 | 3.99626 | 6 |
| let-7i# | 0.027706 | 1 | 6.84317 | -5.84317 | 2.0221 | 2.88965 | 6 |
| miR-144# | 0.033301 | 1 | 6.58335 | -5.58335 | 2.03047 | 2.74978 | 6 |
| miR-590-5p | 0.016849 | 1 | 6.45136 | -5.45136 | 1.66263 | 3.27876 | 6 |
| miR-379 | 0.029727 | 1 | 5.96458 | -4.96458 | 1.75062 | 2.83589 | 6 |
| miR-15a# | 0.009968 | 1 | 5.86073 | -4.86073 | 1.31013 | 3.71011 | 6 |
| miR-331-5p | 0.015028 | 1 | 5.65721 | -4.65721 | 1.38164 | 3.37079 | 6 |
| miR-381 | 0.031337 | 1 | 5.50338 | -4.50338 | 1.61077 | 2.79579 | 6 |
| miR-29c | 0.032274 | 1 | 5.39161 | -4.39161 | 1.58343 | 2.77347 | 6 |
| miR-487a | 0.04072 | 1 | 5.23061 | -4.23061 | 1.62782 | 2.59894 | 6 |
| miR-889 | 0.036123 | 1 | 5.17249 | -4.17249 | 1.55196 | 2.68853 | 6 |
| miR-154# | 0.036646 | 1 | 5.08779 | -4.08779 | 1.52658 | 2.67774 | 6 |
| miR-1227 | 0.011114 | 1 | 5.05771 | -4.05771 | 1.12131 | 3.61873 | 6 |
| miR-28 | 0.00911 | 1 | 4.83541 | -3.83541 | 1.0129 | 3.78657 | 6 |
| miR-185 | 0.008718 | 1 | 4.69541 | -3.69541 | 0.966328 | 3.82418 | 6 |
| miR-589 | 0.022105 | 1 | 4.67657 | -3.67657 | 1.19984 | 3.06422 | 6 |
| miR-570 | 0.027173 | 1 | 4.41485 | -3.41485 | 1.17569 | 2.90455 | 6 |
| miR-130b | 0.014096 | 1 | 4.24412 | -3.24412 | 0.947803 | 3.42278 | 6 |
| miR-1248 | 0.037875 | 1 | 4.11928 | -3.11928 | 1.17574 | 2.65303 | 6 |
| miR-497 | 0.041206 | 1 | 3.96628 | -2.96628 | 1.14524 | 2.5901 | 6 |
| miR-625 | 0.038486 | 1 | 3.77802 | -2.77802 | 1.05186 | 2.64106 | 6 |
| miR-199a | 0.028153 | 1 | 3.75419 | -2.75419 | 0.957175 | 2.87742 | 6 |
| miR-106b | 0.037887 | 1 | 3.69571 | -2.69571 | 1.01618 | 2.65278 | 6 |
| miR-16 | 0.023453 | 1 | 3.63919 | -2.63919 | 0.874445 | 3.01813 | 6 |
| miR-339-3p | 0.047469 | 1 | 3.56397 | -2.56397 | 1.03169 | 2.48521 | 6 |
| miR-150 | 0.000162 | 1 | 3.47924 | -2.47924 | 0.297404 | 8.33627 | 6 |
| miR-708 | 0.031803 | 1 | 3.44824 | -2.44824 | 0.879202 | 2.78461 | 6 |
| miR-10a | 0.012259 | 1 | 3.3818 | -2.3818 | 0.67334 | 3.53729 | 6 |
| miR-942 | 0.010086 | 1 | 3.24606 | -2.24606 | 0.607008 | 3.70021 | 6 |
| miR-200c | 0.022523 | 1 | 3.21972 | -2.21972 | 0.727865 | 3.04963 | 6 |
| miR-30b | 0.033886 | 1 | 2.87222 | -1.87222 | 0.684125 | 2.73666 | 6 |
| miR-151-3p | 0.042699 | 1 | 2.55381 | -1.55381 | 0.606097 | 2.56364 | 6 |
| miR-758 | 0.049001 | 1 | 2.50704 | -1.50704 | 0.612176 | 2.46178 | 6 |
| miR-25 | 0.034763 | 1 | 2.49583 | -1.49583 | 0.550467 | 2.71738 | 6 |
| miR-345 | 0.049837 | 1 | 2.38858 | -1.38858 | 0.566927 | 2.44932 | 6 |
| miR-103 | 0.022021 | 1 | 2.27928 | -1.27928 | 0.417085 | 3.0672 | 6 |
| miR-324-5p | 0.023888 | 1 | 1.85113 | -0.85113 | 0.283342 | 3.00388 | 6 |
